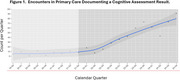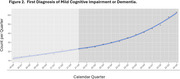# Health system outcomes following an integrated intervention to improve detection of cognitive impairment using a novel training program for primary care

**DOI:** 10.1002/alz70858_101395

**Published:** 2025-12-25

**Authors:** Barak Gaster, Monica Zigman Suchsland, Joshua M. Liao, Sarah McKiddy, Annette L. Fitzpatrick, Basia Belza, Jaqueline G. Raetz

**Affiliations:** ^1^ University of Washington, Seattle, WA, USA; ^2^ University of Texas Southwestern, Dallas, TX, USA

## Abstract

**Background:**

Primary care providers (PCPs) are at the forefront of evaluating cognitive concerns and detecting mild cognitive impairment and dementia, but they generally lack training and tools to do so.

**Method:**

An intervention consisting of education webinars integrated with checklists in the electronic health record (EHR) and a set of exam room tools was developed and implemented across a large primary care system of 14 community‐based clinics (94 PCPs). Outcomes from the EHR included the number of cognitive assessments recorded by PCPs in the EHR and the number of patients who received a new diagnosis of mild cognitive impairment or dementia.

**Result:**

Over two years of the program, the average number of cognitive assessments entered by quarter into the EHR increased from 6.6 to 42.8 (*p* = 0.01). In addition, the average number of new diagnoses of mild cognitive impairment or dementia per quarter increased from 17.0 to 37.8 (*p* = 0.02). See Figures 1 and 2. Referrals to specialty care were reported as being more useful, because they more often included assessments of cognitive function and a review of potential reversible causes of cognitive impairment.

**Conclusion:**

An intervention integrating PCP education with workflow tools increased cognitive testing and diagnoses of mild cognitive impairment and dementia in a large primary care health system. Such change is essential for patients to receive improved care for Alzheimer's disease and related dementias.